# Intragenomic diversity of the V9 hypervariable domain in eukaryotes has little effect on metabarcoding

**DOI:** 10.1016/j.isci.2023.107291

**Published:** 2023-07-12

**Authors:** Olga Flegontova, Julius Lukeš, Aleš Horák

**Affiliations:** 1 Institute of Parasitology, Biology Centre, Czech Academy of Sciences, České Budějovice, Czech Republic; 2 Department of Biology and Ecology, Faculty of Science, University of Ostrava, Ostrava, Czech Republic; 3 Department of Molecular Biology, Faculty of Science, University of South Bohemia, České Budějovice, Czech Republic

**Keywords:** Genetics, Genomics, Computational bioinformatics

## Abstract

Metabarcoding revolutionized our understanding of diversity and ecology of microorganisms in different habitats. However, it is also associated with several inherent biases, one of which is associated with intragenomic diversity of a molecular barcode. Here, we compare intragenomic variability of the V9 region of the 18S rRNA gene in 19 eukaryotic phyla abundant in marine plankton. The level of intragenomic variability is comparable across all the phyla, and in most genomes and transcriptomes one V9 sequence and one OTU is predominant. However, most of the variability observed at the barcode level is probably caused by sequencing errors and is mitigated by using a denoising tool, *DADA2*. The *SWARM* algorithm commonly used in metabarcoding studies is not optimal for collapsing genuine and erroneous sequences into a single OTU, leading to an overestimation of diversity in metabarcoding data. For an unknown reason, *SWARM* inflates diversity of eupelagonemids more than that of other eukaryotes.

## Highlights

• Intragenomic rRNA polymorphism is present in many eukaryotic genomes
• Most of the perceived variability is likely caused by sequencing errors
• *SWARM* overestimates diversity of all eukaryotic taxa, but especially eupelagonemids
• Denoising tools such as *DADA2* provide more accurate estimates of OTU diversity

## Graphical abstract

**Figure:**
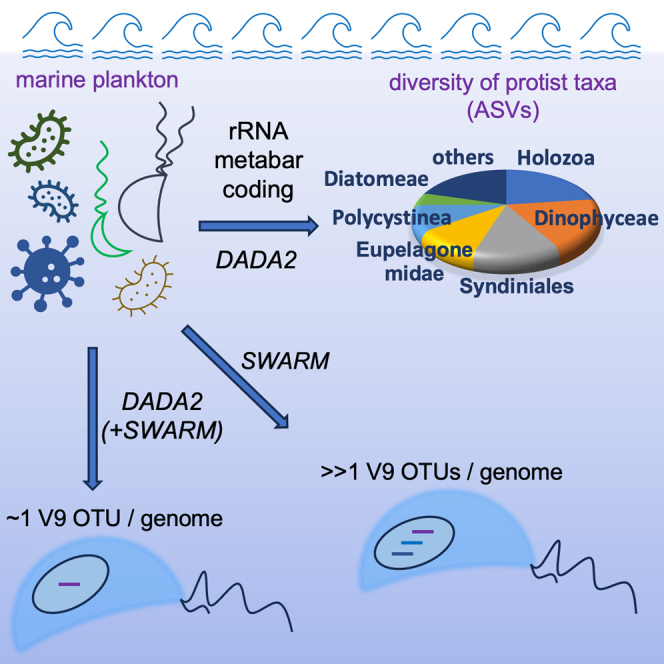

## Introduction

The combination of DNA barcoding and second-generation sequencing technologies (commonly known as metabarcoding) has revolutionized our understanding of microbial diversity over the past two decades.^1^ In addition to several undeniable advantages, metabarcoding also has inherent limitations, mostly related to the PCR amplification step.^2^^,^^3^^,^^4^^,^^5^ A putatively important but poorly documented problem of metabarcoding is the intragenomic variability of molecular barcodes. Possible examples of this phenomenon have recently been reported in radiolarians,^6^^,^^7^ ciliates,^8^^,^^9^ and diplonemids.^10^ The latter have been described as the most diverse protist phylum in marine plankton, accounting for up to 17% of operational taxonomic units (OTUs) defined in a global dataset.^11^ However, Mukherjee et al.^10^ examined the intragenomic variability of diplonemid 18S rRNA genes and argued that their diversity may be overestimated in metabarcoding studies.

The availability of genomic data for a growing number of eukaryotic groups allowed us to assess the extent of intragenomic variability of the V9 region of the 18S rRNA gene in major planktonic eukaryotes and its implications for the existing global metabarcoding datasets.^12^^,^^13^ We also used a tool for removing sequencing errors (denoising) in amplicon data^14^ and examined the effects of denoising and OTU definition strategies on diversity estimates for diplonemids (mainly eupelagonemids) and other key marine planktonic eukaryotes. Although similar methodological comparisons are available for a range of organisms,^9^^,^^15^^,^^16^^,^^17^^,^^18^^,^^19^ no such results have been published for the most abundant and diverse marine protist taxa.

## Results

### Exploring apparent intragenomic sequence variability at the level of V9 barcodes

**Figure 1:**
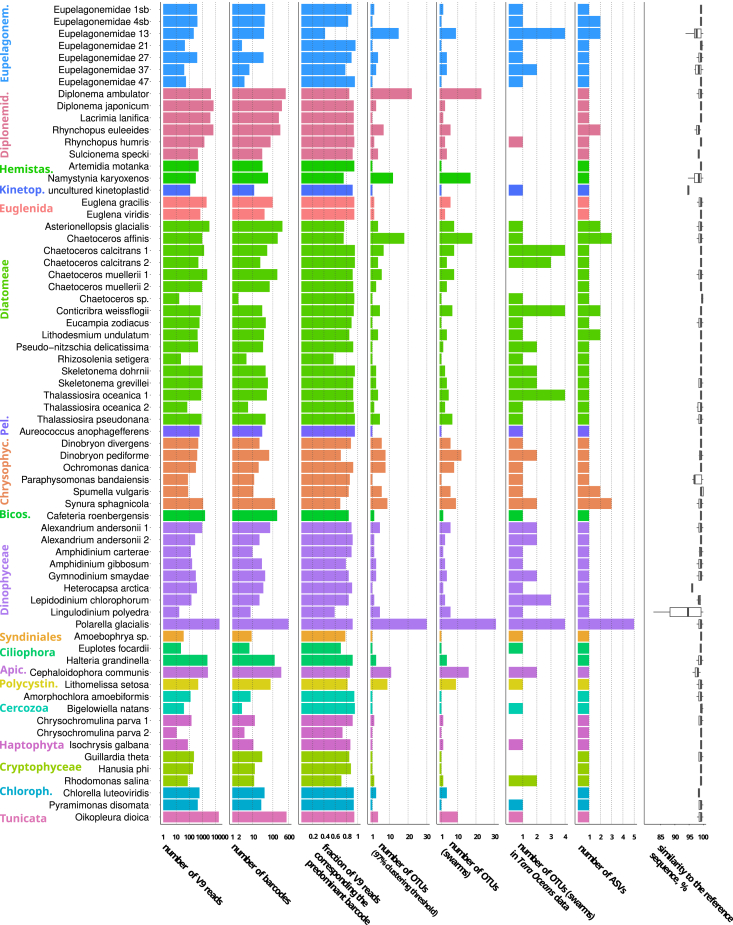
Key statistics related to V9 reads and OTUs extracted from the studied genomes/transcriptomes The following information is shown: number of V9 reads per genome/transcriptome (in log_10_ scale); number of V9 barcodes (in log_10_ scale); fraction of V9 reads corresponding to a barcode most abundant in the genome/transcriptome; number of OTUs generated by the centroid clustering approach with a 97% identity threshold; number of OTUs generated by the *SWARM* approach; number of OTUs ("swarms") per genome/transcriptome that were found in the *Tara Oceans* metabarcoding data; number of ASVs inferred by the *DADA2* algorithm; boxplots summarizing identity of barcodes found in the genomes/transcriptomes to best hits in the reference taxonomic database. The boxplots show the following values: median, 25^th^ and 75^th^ percentiles (the box), the largest value within 1.5× interquartile range above the 75^th^ percentile (the upper whisker), the smallest value within 1.5× interquartile range below the 25^th^ percentile (the lower whisker). Taxa are labeled and color-coded. The same statistics are shown in Table S2. The statistics were calculated following contamination removal described in the text for V9 barcodes, OTUs and ASVs. Abbreviations: Apic., Apicomplexa; Bicos., Bicosoecida; Chrysophyc., Chrysophyceae; Chloroph., Chlorophyta; Diplonemid., Diplonemidae; Eupelagonem., Eupelagonemidae; Hemistas., Hemistasiidae; Kinetop., Kinetoplastea; Pel., Pelagophyceae; Polycystin., Polycystinea.

For each genome/transcriptome, we extracted full-length sequences of the V9 18S rRNA variable region (ranging from 104 to 137 nucleotides [nt]) from raw reads using an 80% similarity cutoff against sequences in the PR2 database.^20^ The extracted sequences of V9 region (barcodes) with additional information are listed in Table S1. Two genomes were incorrectly annotated at the phylum level in the Short Read Archive: a diatom and a chlorophyte were sequenced instead of dinoflagellates. Twenty-six of 68 genomes/transcriptomes were found to be contaminated with taxonomically distant organisms belonging to other protist phyla (Figure S1). Our results suggest that these contaminations can be removed by discarding barcodes that have <80% identity to the most abundant V9 barcode in the genome/transcriptome, as almost all of these barcodes have the best matches to other eukaryotic phyla in the reference database (Figure S1). Following this procedure, each genome/transcriptome contained at least two distinct V9 barcodes (from 2 to 600 barcodes per genome/transcriptome, median = 34.5). The number of barcodes and other summary statistics are shown in Figure 1 and Table S2.

The number of V9 barcodes per genome/transcriptome depends on the number of extracted V9 reads (Figure S2A), which is a function of both sequencing depth and the number of 18S rRNA gene copies in the genome. The V9 barcode variability we observe within genomes/transcriptomes may be caused by 1) intragenomic polymorphisms, which we analyze here, 2) sequencing errors, and 3) contamination with organisms belonging to the same phylum (such type of contamination was not removed by our procedure). To mitigate the former two problems, we either grouped barcodes into OTUs defined in two different ways and/or applied to the extracted V9 reads a denoising algorithm designed to correct sequencing errors in the amplicon data (*DADA2*).^14^ In addition to two types of OTU counts and counts of denoised barcodes (amplicon sequence variants or ASVs), another metric we used was the fraction of V9 reads belonging to a barcode most abundant in a given genome/transcriptome. None of these four metrics showed a dependence on the number of extracted V9 reads, as seen for barcodes (Figures S2A–S2E), and two metrics gave highly correlated results: centroid-based sequence clustering with a 97% identity threshold and the *SWARM* OTU definition algorithm^21^ (Figure S2F). For this reason, we consider only one OTU definition algorithm in the following text, namely *SWARM*.

**Figure 2:**
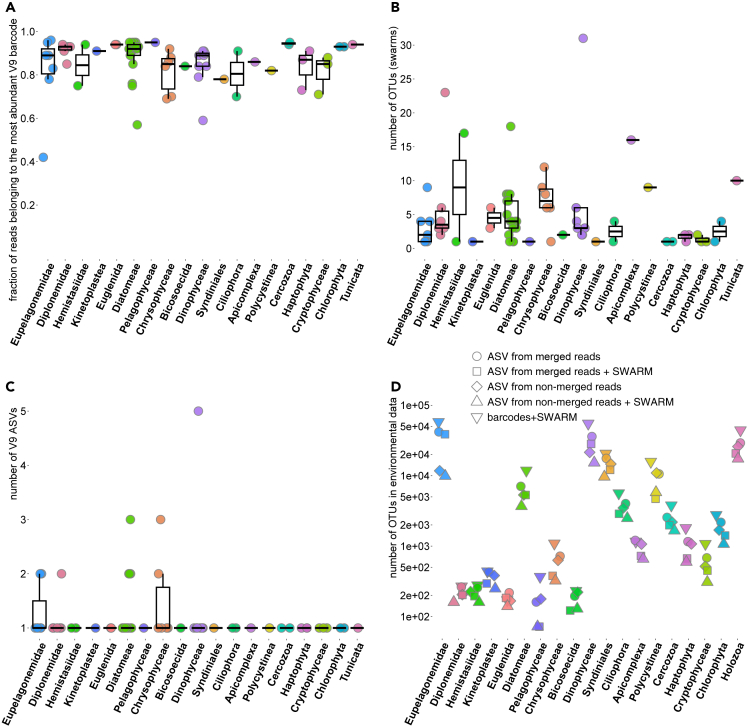
Comparison of 19 eukaryotic taxa according to three metrics of (supposed) intragenomic sequence variability (A–C) and according to the number of ASVs or OTUs in marine plankton estimated with three different methods (D). In panels A–C, each dot represents a genome/transcriptome arranged according to the proportion of reads belonging to the most abundant V9 barcode in that genome/transcriptome (A); or according to the number of OTUs (swarms) (B) or ASVs (C) found in that genome/transcriptome following contamination removal. The boxplots summarize distributions of these metrics within the taxa. In panel D, the number of ASVs or OTUs in marine plankton (the *Tara Oceans* data) is shown for the same set of taxa in logarithmic scale. ASVs/OTUs were inferred using five methods: *DADA2* on merged V9 amplicon reads (following the same protocol as applied to the genomic data in panel C); *DADA2* on non-merged V9 amplicon reads (following the protocol from [9, 10]); *SWARM* OTUs derived from V9 barcodes; ASVs generated using both *DADA2* protocols were optionally clustered with *SWARM*. The boxplots in panels A–C show the following values: median, 25^th^ and 75^th^ percentiles (the box), the largest value within 1.5× interquartile range above the 75^th^ percentile (the upper whisker), the smallest value within 1.5× interquartile range below the 25^th^ percentile (the lower whisker).

Looking at the proportion of V9 reads belonging to a barcode that is most abundant in a given genome/transcriptome, we find that this statistic varies widely within taxa but is relatively uniform across taxa (Figures 1, 2A, and Table S2). In most cases, there is a single predominant barcode in a genome/transcriptome (median read fraction = 0.9), although there are several outlier genomes/transcriptomes within Eupelagonemidae, Diatomeae, Dinophyceae, Chrysophyceae, and other clades. The most striking case is the eupelagonemid single-cell genome number 13,^22^ where two barcodes with a pairwise similarity of 98.4% had almost equal abundance (93 and 86 reads, Table S1).

### Only one OTU per genome is overwhelmingly found in environmental metabarcoding data

**Figure 3:**
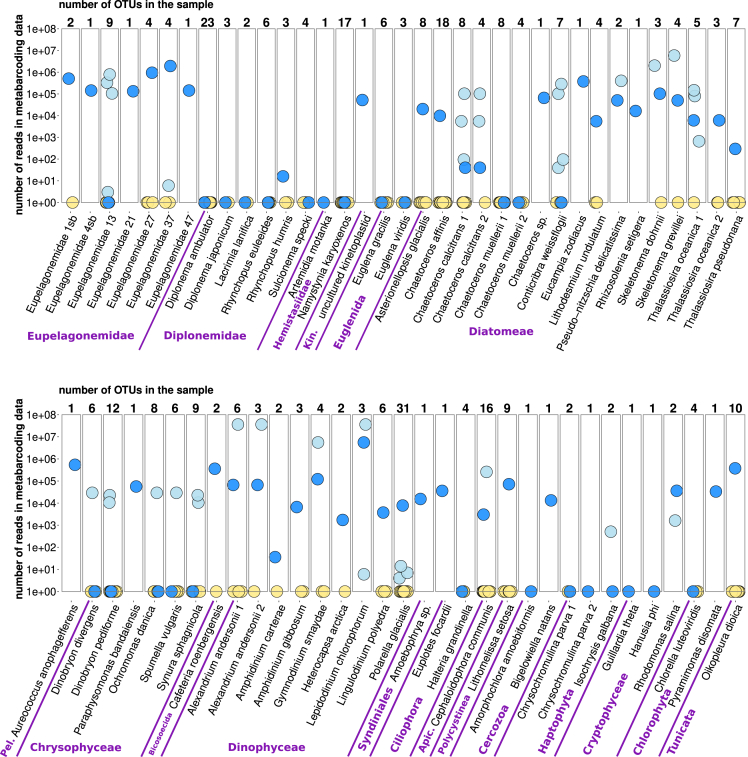
Abundance in metabarcoding data of *SWARM* OTUs (derived from V9 barcodes) found in the 68 genomes/transcriptomes studied Each dot represents a single V9 OTU. The total count of OTUs in genomes/transcriptomes is shown above the bars. The logarithmic y axis shows OTU abundance in the *Tara Ocean* metabarcoding dataset [9–10]. OTUs with no reads found in the environment are arranged at the bottom of the scale. OTUs are color-coded in the following way: predominant (supposedly intragenomic) OTUs are colored in dark-blue; minor intragenomic OTUs which were found in the metabarcoding dataset are colored in light blue; minor OTUs which were not found in the metabarcoding dataset are colored in yellow. Abbreviations: Apic., Apicomplexa; Kin., Kinetoplastea; Pel., Pelagophyceae.

In the following, we focus on *SWARM* OTUs, which we refer to as “swarms” for brevity. The genomes/transcriptomes also vary greatly within taxa according to the number of (supposedly intragenomic) swarms, but there are no clear differences in the number of OTUs between taxa (Figures 1, 2B, and Table S2). Median number of swarms per genome/transcriptome is three; 18 of 68 genomes/transcriptomes contain only one OTU; the highest number of OTUs (31) was detected in a dinophyte *Polarella glacialis*. To understand how this putative intragenomic OTU variability is reflected in the metabarcoding studies, we quantified the abundance of swarms derived from the 68 genomes/transcriptomes in a V9 metabarcoding dataset of global marine eukaryotic plankton (*Tara Oceans*^12^^,^^13^). For 18 genomes/transcriptomes, no matching V9 OTUs were found in the *Tara Oceans* data, and 13 genomes/transcriptomes detected in the *Tara Oceans* data contained only one intragenomic OTU (Figure 3). Therefore, these two groups are not informative for our purposes.

For 15 genomes/transcriptomes, only the most abundant intragenomic OTU was found in the *Tara Oceans* data, so their presumed intragenomic V9 variability is not reflected in this metabarcoding study (Figure 3). For 19 genomes/transcriptomes, minor intragenomic OTUs were more abundant in the *Tara Oceans* metabarcoding data (in some cases by several orders of magnitude) than the corresponding main intragenomic OTUs (Figure 3). These results suggest that either intragenomic variability or sequencing errors generate sequences that match V9 regions of more abundant species, or that minor intragenomic OTUs actually represent contamination with closely related organisms. We found only three examples where (putative) intragenomic V9 variability (expressed as swarms) is reflected in metabarcoding data: the dinophyte *P. glacialis*, the cryptophyte *Rhodomonas salina*, and the eupelagonemid cell 37 (Figure 3). In these three genomes, both predominant and minor OTUs are detected in the *Tara Oceans* data, with the former more abundant than the latter in the metabarcoding data.

### Most of the observed intragenomic polymorphism in the V9 region is caused by sequencing errors

Next, we applied the *DADA2* denoising algorithm to V9 reads extracted from the examined 68 genomes/transcriptomes. The results are presented in Table S3. In total, 130 ASVs had higher than 80% similarity to the best hits in the reference sequence database (the same threshold was used for barcodes described previously). Thus, the denoising procedure reduced the number of barcodes by 41-fold, suggesting that the intragenomic sequence diversity we observe at the barcode level is largely due to sequencing errors. We also found that 89% of ASVs that diverge from a predominant ASV in a genome/transcriptome (sequence similarity of 90% or less) have 100% matches to sequences in the reference database, while only 20% of less divergent ASVs (sequence similarity from 90% to 100%) have these ideal matches in the reference database. Therefore, we assume that the intragenomic ASVs that have a sequence similarity of less than 90% to the most abundant ASV are not truly intragenomic and represent contamination. In the case of less divergent intragenomic ASVs (sequence similarity from 90% to 100%), both contamination and true intragenomic variability remain plausible. We consider these latter cases as intragenomic variability in the following.

After this round of contamination removal, only 10 of 68 genomes/transcriptomes contained more than one ASV, ranging from 2 to 5 (Figures 1, 2C, and Table S2): those of chrysophytes (2 of 6 genomes/transcriptomes), diatoms (4 of 17), a dinophyte (1 of 9), a diplonemid (1 of 6), and eupelagonemids (2 of 7). Considering that supposedly true intragenomic variability is rare, it is difficult to judge whether a taxon is outstanding according to the level of intragenomic variability in the V9 region. The minor and major intragenomic ASVs mostly do not cluster together (in the cases of 7 of 10 genomes/transcriptomes) using the *SWARM* approach in the context of the *Tara Oceans* V9 data. For all 10 genomes/transcriptomes with multiple ASVs, only a single ASV was found in the *Tara Oceans* data (Table S4), again suggesting that intragenomic V9 sequence variability is not reflected in the metabarcoding studies.

**Figure 4:**
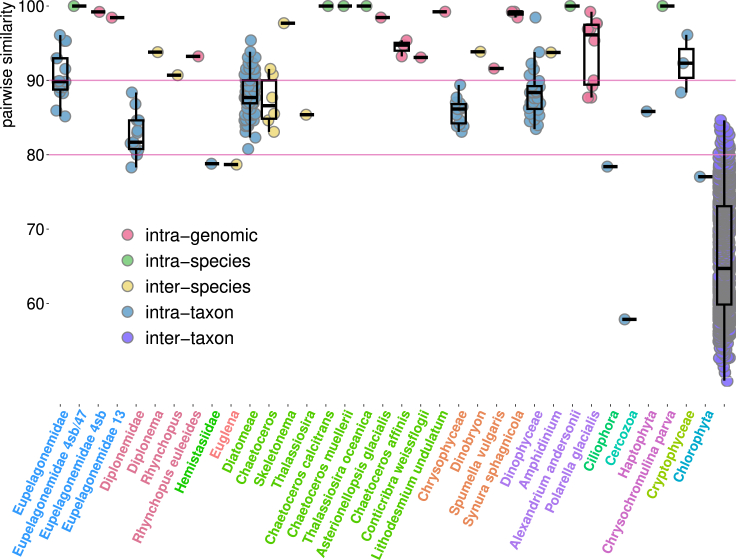
Comparing sequence divergence in the V9 region, measured as pairwise similarity of aligned sequences, at various levels: intragenomic (following contamination removal), intra-species, inter-species (within a genus), intra-taxon, and inter-taxon These levels are color-coded according to the legend. Species, genera, and higher taxa are labeled on the *x* axis, and distributions of sequence similarity are summarized using boxplots. The boxplots show the following values: median, 25^th^ and 75^th^ percentiles (the box), the largest value within 1.5× interquartile range above the 75^th^ percentile (the upper whisker), the smallest value within 1.5× interquartile range below the 25^th^ percentile (the lower whisker).

Outcomes of further inspection of intragenomic V9 sequence variability in these 10 genomes/transcriptomes, as well as within and between species and higher taxonomic units are shown in Figure 4. There are five pairs of genomes/transcriptomes of the same species in our dataset. In all these cases, there is only a single ASV per genome/transcriptome, and it is identical within the species (Figure 4). Levels of intragenomic and inter-species V9 sequence divergence (measured as pairwise sequence similarity) largely overlap in our limited dataset, and there is even an overlap between intragenomic and intra-taxon sequence divergence levels (Figure 4). This suggests that discriminating genuine variability from contamination in these 10 species is difficult if not impossible.

### The *SWARM* OTU definition algorithm overestimates diversity unequally across eukaryotic lineages

Since we found that an approach based on the *SWARM* OTU definition is not optimal for clustering genuine sequences and those derived from sequencing errors, we attempted to assess whether diversity estimates for common marine eukaryotic taxa in the *Tara Oceans* data-based studies,^12^^,^^13^ which relied on the *SWARM* method, are inflated. We applied this method following the original publications, and also two versions of the *DADA2* algorithm: the original protocol where unmerged amplicon reads with trimmed primers and adapters served as input and a modified protocol where merged reads served as input. The latter was included for compatibility with our results presented previously, since we could not start the *DADA2* workflow with unmerged V9 reads in the analyzed set of 68 genomes/transcriptomes, and used full-length V9 regions extracted from genomic reads instead. Thus, we compared five methods for estimating diversity of eukaryotic taxa: *SWARM* OTUs, two ASV protocols, and both ASV protocols followed by *SWARM* clustering. Complete results of this analysis are shown in Table S5, and results for selected taxa are presented in Figure 2D. In most cases (77% of taxa), the *SWARM* method produced the highest OTU counts, while the classic *DADA2* protocol produced the lowest OTU counts (for 74% of taxa) (Table S5). *SWARM* clustering applied to outcomes of the classic *DADA2* protocol also tends to generate the lowest OTU counts among the two “ASV + *SWARM*” protocols (for 64% of taxa) (Table S5).

Surprisingly, the extent of diversity overestimation differs across taxa. The median ratio of OTU counts found by the *SWARM* method compared to the classic *DADA2* protocol is just 1.5 for 70 taxa (Table S5), but there are some outlying taxa whose diversity is much more affected by the OTU definition and denoising protocols. Eupelagonemids (and a related diplonemid clade DSPD II) are the most striking case: this is the most diverse eukaryotic clade in marine plankton according to the *SWARM* approach,^11^^,^^23^ but its OTU richness decreases 5-fold according to the classic *DADA2* protocol, turning these flagellates into the fourth most diverse taxon after holozoans, dinophytes, and syndinians (Figure 1D; Table S5). When the classic *DADA2* protocol is combined with the *SWARM* algorithm, eupelagonemids become the third most diverse taxon after holozoans and dinophytes. In general, application of the *SWARM* algorithm to ASV data reduces the number of OTUs by 1.4-fold (median value for 70 taxa) (Table S5).

## Discussion

Given the (at least) theoretical impact of intragenomic variability on metabarcoding in general, it is surprising that no systematic effort was dedicated to uncover its extent and effect. Gong and Marchetti^24^ aimed to correct the estimates of rRNA copy numbers based on the V4 region among the representatives of main eukaryotic phytoplankton taxa and discussed its effect on abundance estimates relying on the metabarcoding data. Unfortunately, they did not address the intragenomic diversity of V4 metabarcodes. Multiple V9 OTUs defined using either the *SWARM* or *DADA2* algorithms were found in clonal cultures of 29 ciliate species.^9^ In addition, several OTUs of the V9 and V4 regions were found in samples derived from individual radiolarian cells.^6^^,^^7^ The latter study shows that despite the relatively low error rates associated with this sequencing platform, the high sequencing depth of Illumina-based metabarcoding can introduce considerable amount of noise (particularly due to tag-jumping and cross-contamination). Given the fact that the vast majority of recent metabarcoding studies use the Illumina technology, special caution should be taken when interpreting these results. Although we focus primarily on genomic and transcriptomic data, the level of contamination present there (found in at least 26 out of 68 genomes/transcriptomes) is surprising.

It has also been shown that conceptually different OTU definition protocols such as *SWARM*, *DADA2*, and *VSEARCH* greatly overestimate the species diversity of land plant communities based on metabarcoding of the nuclear rRNA internal transcribed spacer region 2 (ITS2).^17^ This well-documented overestimation of species richness has been attributed to sequencing errors, barcode amplification artifacts, and intragenomic and intra-population sequence variability.^4^^,^^5^^,^^25^ Our results on a range of eukaryotic genomes and transcriptomes suggest that the bulk of sequence variability observed at the barcode level is in fact caused by sequencing errors. The *SWARM* algorithm is often unable to collapse genuine sequences and erroneous sequences derived from them into a single OTU, leading to overestimation of diversity in the metabarcoding data. One of the interesting implications of this observation could be found for the rare biosphere concept,^26^^,^^27^^,^^28^ where denoising has typically been applied only to the pyrosequencing data.^29^^,^^30^ However, if intragenomic polymorphism and sequencing errors are relevant also for the Illumina-based metabarcoding data, do they artificially increase observed diversity of (mostly) rare OTUs? We found some evidence of intragenomic sequence variability in the V9 region in both *SWARM* OTUs and ASVs, but species demonstrating this phenomenon are distributed sporadically across taxa (Figures 1 and 2). Since only one V9 barcode, *SWARM* OTU, or ASV is highly prevalent in nearly all genomes/transcriptomes (Figure 2A), this intragenomic variability does not result in inflated OTU counts in the *Tara Oceans* metabarcoding dataset (Figure 3).

Our study focuses on the V9 variable domain, shorter and generally more conserved as compared to the V4 barcode, which was found to reflect more realistically the diversity of the full-length rRNA.^31^^,^^32^ However, this is not a universally accepted view^33^^,^^34^^,^^35^ and the performance of specific barcodes could be influenced by various factors.^36^ Since one of our groups of interest, diplonemids, are severely underrepresented in the V4-based studies^23^ due to their long V4 region (∼650 bp vs. ∼450 bp typical for most eukaryotic phyla), here we focus only on the V9 data. However, we acknowledge that analysis of the V4 variable domain may lead to slightly different results (although the nature of these differences is unpredictable and may differ among individual eukaryotic clades).

According to *SWARM* OTUs in the V9 metabarcoding data, eupelagonemids represent the most diverse eukaryotic group in marine plankton,^11^^,^^23^ and they did not demonstrate outstandingly high levels of intragenomic V9 sequence variability in our study. For some reason, the *SWARM* algorithm overestimates the diversity of this taxon in the *Tara Oceans* data much more than that of nearly all other eukaryotic taxa (Figure 2D and Table S5). This effect may be attributed to an uneven distribution of sequencing errors in the V9 region across taxa or to the behavior of the *SWARM* algorithm itself. These observations are relevant for assessing diversity of the rare biosphere: we showed that the level of diversity overestimation due to the *SWARM* algorithm differs substantially across eukaryotic taxa, but taxa also substantially differ in their OTU abundance distributions, some of them having very long “rare” tails.^1^ For example, it is known that the abundance distribution of eupelagonemid OTUs has this long “rare” tail.^23^ Thus, diversity estimates and taxonomic profiles for the rare biosphere in marine plankton are expected to differ substantially depending on whether *SWARM*, *DADA2*, or other protocols are used.

### Limitations of the study

We are aware of the following limitations of our study. The first is related to the availability of genomic data for planktonic eukaryotes. Due to sampling biases, our dataset is rich for some lineages such as diplonemids (data largely available due to our own efforts) and photosynthetic stramenopiles. On the other hand, other relevant taxa such as ciliates, various rhizarian lineages, or dinoflagellates (including “marine alveolates” also known as syndinians) are poorly represented. This could affect the overall picture of intragenomic sequence variability in the V9 18S rRNA region presented in Figures 1, 2, 3, and 4. In addition, one of our major findings is that most of the V9 polymorphism found in genomic and transcriptomic data is due to sequencing errors. This is based on the results of the *DADA2* denoising algorithm, whose performance has been tested mainly on amplicon, not on shotgun sequencing, data and is highly dependent on sequencing depth. Therefore, we cannot exclude the possibility that some of the genuine intragenomic variability was incorrectly removed by denoising.

## STAR★Methods

### Key resources table

**Table:** 

REAGENT or RESOURCE	SOURCE	IDENTIFIER
**Software and algorithms**		

*SWARM* v.2	Mahé et al.^21^	https://github.com/torognes/swarm
*DADA2*	Callahan et al.^14^	https://benjjneb.github.io/dada2/
*GGSEARCH36* (the *FASTA* package)	N/A	ftp://ftp.ebi.ac.uk/pub/software/unix/fasta

**Other**		

Reads of 68 eukaryotic genomes and transcriptomes	multiple publications	See our Table S2 for details
18S rRNA gene metabarcoding data (*Tara Oceans*)	de Vargas et al., Ibarbalz, et al.^12^^,^^13^	See our Table S6 for details
Taxonomically annotated database of V9 18S rRNA regions	the *Tara Oceans* project	www.taraoceans.sb-roscoff.fr/EukDiv/data/Database_W2_v9_pr2.fasta
PR2 18S rRNA sequence database (release 4.14.0)	Guillou et al.^20^	www.pr2-database.org

### Resource availability

#### Lead contact

Further information and requests for resources should be directed to and will be fulfilled by the lead contact, Olga Flegontova (olga@paru.cas.cz).

#### Materials availability

This study did not generate new unique materials.

### Method details

#### A reference database for the V9 region of the 18S rRNA gene

A copy of the *PR2* 18S rRNA sequence database^20^ was downloaded from www.pr2-database.org (release 4.14.0). Reference 18S rRNA sequences were divided into several large taxonomic groups and were aligned within these groups using *MAFFT* v. 7.490^37^ under the following settings: --maxiterate 1000 --localpair. Full-length V9 region sequences were extracted by mapping universal V9 eukaryotic primers “389F” (5′-TTGTACACACCGCCC-3′) and “1510R” (5′-CCTTCYGCAGGTTCACCTAC-3′)^38^ on the alignments; up to 5 mismatches in the primer sequences were allowed. We ensured that complete V9 regions were extracted and that V9 sequences for no phylum were missed by comparing them to a database of V9 regions prepared by the *Tara Oceans* team (www.taraoceans.sb-roscoff.fr/EukDiv/data/Database_W2_v9_pr2.fasta).

#### Extraction of V9 region sequences from 68 genomes/transcriptomes

We analyzed intragenomic diversity of the V9 region of the 18S rRNA gene in 68 published genomes/transcriptomes belonging to nineteen taxonomic groups (Table S2) covering various branches of the eukaryotic tree of life. Species selected for the study were detected in the marine plankton in a global metabarcoding study by the *Tara* consortium^12^^,^^13^ or were related to those species. Raw reads for 68 genomes or transcriptomes were downloaded from the Short Read Archive (SRA) or from the European Nucleotide Archive (ENA). We considered transcriptomes for analysis only if the genome of a given species was not present in SRA or ENA. Information on the 68 genomes/transcriptomes is shown in Table S2.

Trimming of Illumina adapter sequences was performed using *BBDUK* (the *BBTOOLS* package, https://sourceforge.net/projects/bbmap/) under the default settings. The trimmed reads were merged using *BBMERGE* (the *BBTOOLS* package^39^) under the default settings. Merged and non-merged reads having average Phred quality below 20 were removed using *BBDUK*.

Processed reads (merged and non-merged) were aligned using *BLAST* v. 2.12.0+ against the V9 reference database (see above) under the following settings: -evalue 1 -max_target_seqs 1. From the blast results, only sequences aligned from the first to the last nucleotide of the reference V9 region (104-137 nucleotides long) were extracted. V9 sequences containing undetermined ("N") nucleotides were deleted using *BBDUK* (the *BBTOOLS* package), and the remaining V9 sequences were dereplicated into barcodes using *VSEARCH* v. 2.7.1^40^ under the default settings.

#### Processing the *Tara Oceans* V9 metabarcoding dataset

To explore how the supposed intragenomic V9 variability might be reflected in a metabarcoding study, we analyzed the *Tara Oceans* V9 metabarcoding dataset (Table S6; de Vargas et al., 2015^12^, Ibarbalz et al., 2019^13^). Primer sequences were removed using *CUTADAPT* v. 1.15^41^ under the following settings: --no-indels, --discard-untrimmed, --minimumlength 50, --overlap 4, -e 0.2, -a TTGTACACACCGCCC... GTAGGTGAACCTGCRGAAGG, -A CCTTCYG-CAGGTTCACCTAC... GGGCGGTGTGTACAA. Reads were then merged using *BBMERGE* under the default settings. Using *BBDUK*, merged reads containing undetermined bases (Ns) were filtered out and reads having average Phred quality below 20 were discarded. Cleaned reads were dereplicated into barcodes using *VSEARCH* v. 2.7.1^40^ under the default settings.

#### Denoising V9 reads using the *DADA2* algorithm

Sequences of the V9 region extracted from 68 genomes/transcriptomes using *BLAST*, which had already been subjected to primer removal and merging (see above), were pooled together and served as a starting point for processing with *DADA2*. In the case of the *Tara Oceans* V9 metabarcoding dataset, two protocols were used: 1/ the original protocol where non-merged amplicon reads with trimmed primers and adapters served as input (Callahan et al., 2016^14^; https://benjjneb.github.io/dada2/tutorial.html); 2/ a modified protocol where, for compatibility with the processing of V9 regions extracted from 68 genomes/transcriptomes, merged reads served as input. Due to the large size of the *Tara Oceans* V9 metabarcoding dataset (420 GiB in 1,258 samples), machine learning that generates sequencing error models was carried out on each sample separately, in contrast to V9 regions from 68 genomes/transcriptomes which had to be pooled for this step. When the *DADA2* protocol was started from merged reads, the *fastqFilter* function (maxN=0, maxEE=2) was applied first, followed by the *learnErrors*, *dada*, and *removeBimeraDenovo* functions (method=’consensus’) from the *DADA2* R package. In the case of the original *DADA2* protocol, the *filterAndTrim* function (truncLen=c(80,80), maxN=0, maxEE=c(2,2), truncQ=2) was applied first, followed by the *learnErrors* and *dada* functions applied separately to forward and reverse reads, then by the *mergePairs* and *removeBimeraDenovo* functions (method=’consensus’). To check the efficiency of error model inference we plotted observed error rates for each consensus quality score (Figures S3–S6). Estimated error rates after convergence of the machine-learning algorithm looked reasonable in all cases: for forward (Figure S3), reverse (Figure S4), and merged reads (Figure S5) of the *Tara Oceans* V9 metabarcoding dataset and for pooled V9 regions extracted from the 68 genomes/transcriptomes (Figure S6). We conclude that all the *DADA2* protocols tested here are applicable to the data analyzed in this study.

#### Taxonomical annotation

Barcodes and amplicon sequence variants (ASVs, output of *DADA2* protocols) were taxonomically annotated using the *GGSEARCH36* software from the *FASTA* package (ftp://ftp.ebi.ac.uk/pub/software/unix/fasta) under the following settings: -m 8 -d 0 -b 1 -E 10 -w 199. As references for the annotation, the V9 reference database was used (see above).

#### OTU inference using the *SWARM* method

OTUs were inferred based on V9 barcodes or V9 ASVs using *SWARM* v.2 (Mahé et al., 2015) with the default settings (-d 1 -f -z). For generation of “swarm OTUs”, only those *Tara Oceans* barcodes or ASVs were taken that occurred in more than one sample and had an abundance of more than two reads. For generating swarm OTUs in the case of the 68 genomes/transcriptomes, V9 barcodes/ASVs derived from them were combined with the *Tara Oceans* V9 barcodes/ASVs.

#### Centroid-based clustering for OTU inference

Clustering was performed at a 97% similarity level on V9 barcodes separately for each genome/transcriptome using *VSEARCH* v. 2.7.1^40^ under the following settings: --id 0.97 --cluster_size –mothur_shared_out.

### Quantification and statistical analysis

All statistical analyses and plotting were performed using R v. 4.2.3. Pairwise sequence similarity was calculated using the *pairwiseAlignment* function (the *Biostrings* R package) under the following settings: substitutionMatrix=nsm (match=1, mismatch=-3, baseOnly=TRUE, type=’DNA’), gapOpening=5, gapExtension=2, type=’global’. Interactions between different variables such as number of V9 reads, number of V9 barcodes, number of V9 OTUs, number of V9 ASVs per genome/transcriptome, and fraction of reads belonging to the most abundant V9 barcode in the genome/transcriptome (Figure S2) were explored using generalized additive models (GAM) as implemented in the *mgcv* v. 1.8-38 R package. We fitted GAMs (y ∼ s(x)) based on the gamma distribution that is suitable for positive continuous variables (family=Gamma(link=’identity’), method=’REML’). In the case of the fraction of reads belonging to the most abundant V9 barcode, we fitted GAMs (y ∼ s(x)) based on the beta distribution that is suitable for variables ranging between 0 and 1 (family=betar(eps=0.00001)).

## Data Availability

• This paper analyzes existing, publicly available data; see the accession numbers listed in Tables S2 and S6. See key resources table for a list of publicly available software packages used for data analysis. • Any additional information required to reanalyze the data reported in this paper is available from the lead contact upon request.

## Supplemental information

Document S1. Figures S1–S6

Table S1. List of all V9 18S rRNA barcodes extracted from the 68 genomes/transcriptomes, related to Figure 1

Table S2. Data sources for the genomes/transcriptomes used in the study and statistics related to V9 reads and ASVs/OTUs, related to Figure 1

Table S3. List of ASVs inferred in the 68 genomes/transcriptomes using the *DADA2* de-noising algorithm, related to Figure 2

Table S4. Abundance of (supposedly) intra-genomic ASVs and *SWARM* OTUs derived from them in the *Tara Oceans* metabarcoding data, related to Figure 2D

Table S5. Diversity estimates for eukaryotic phyla in marine plankton based on the *Tara Oceans* V9 metabarcoding data, related to Figure 2D

Table S6. A list of SRA accession numbers for the *Tara Oceans* V9 metabarcoding dataset used in this study, related to Figure 2D
