# Supplementary material for: Intragenomic diversity of the V9 hypervariable domain in eukaryotes has little effect on metabarcoding
Source: iScience. 2023 Jul 12;26(8):107291. doi: 10.1016/j.isci.2023.107291 (PMC10404988; doi:10.1016/j.isci.2023.107291)
Supplement: Document S1. Figures S1–S6 [file mmc1.pdf]

iScience, Volume *26*

Supplemental information

Intragenomic diversity of the V9 hypervariable domain in eukaryotes has little effect on metabarcoding

Olga Flegontova, Julius Lukeš, and Aleš Horák

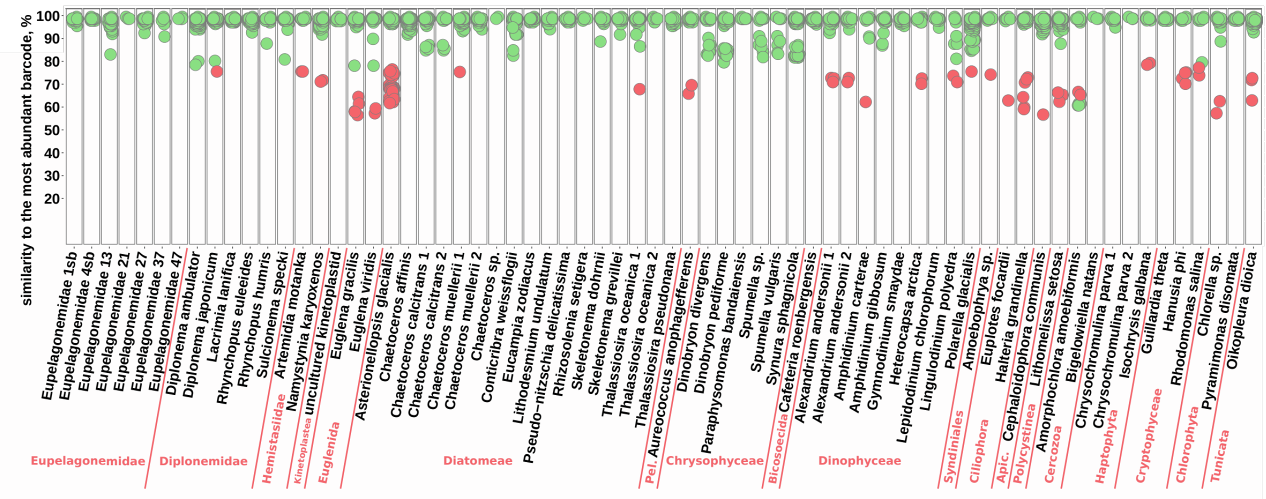

Figure S1. Divergence of (supposedly) intra-genomic V9 barcodes from the most abundant V9 barcode in the genome/transcriptome. Each dot represents a V9 barcode. Similarity to the most abundant barcode in the genome/transcriptome is shown on the y-axis in percent. Barcodes whose taxonomic annotation at the phylum level matches that of the most abundant barcode are colored in green, and those with mismatched phylum annotation are colored in red. The latter group of barcodes was removed from downstream analyses as obvious contamination. Abbreviations: Apic. – Apicomplexa; Pel. – Pelagophyceae.

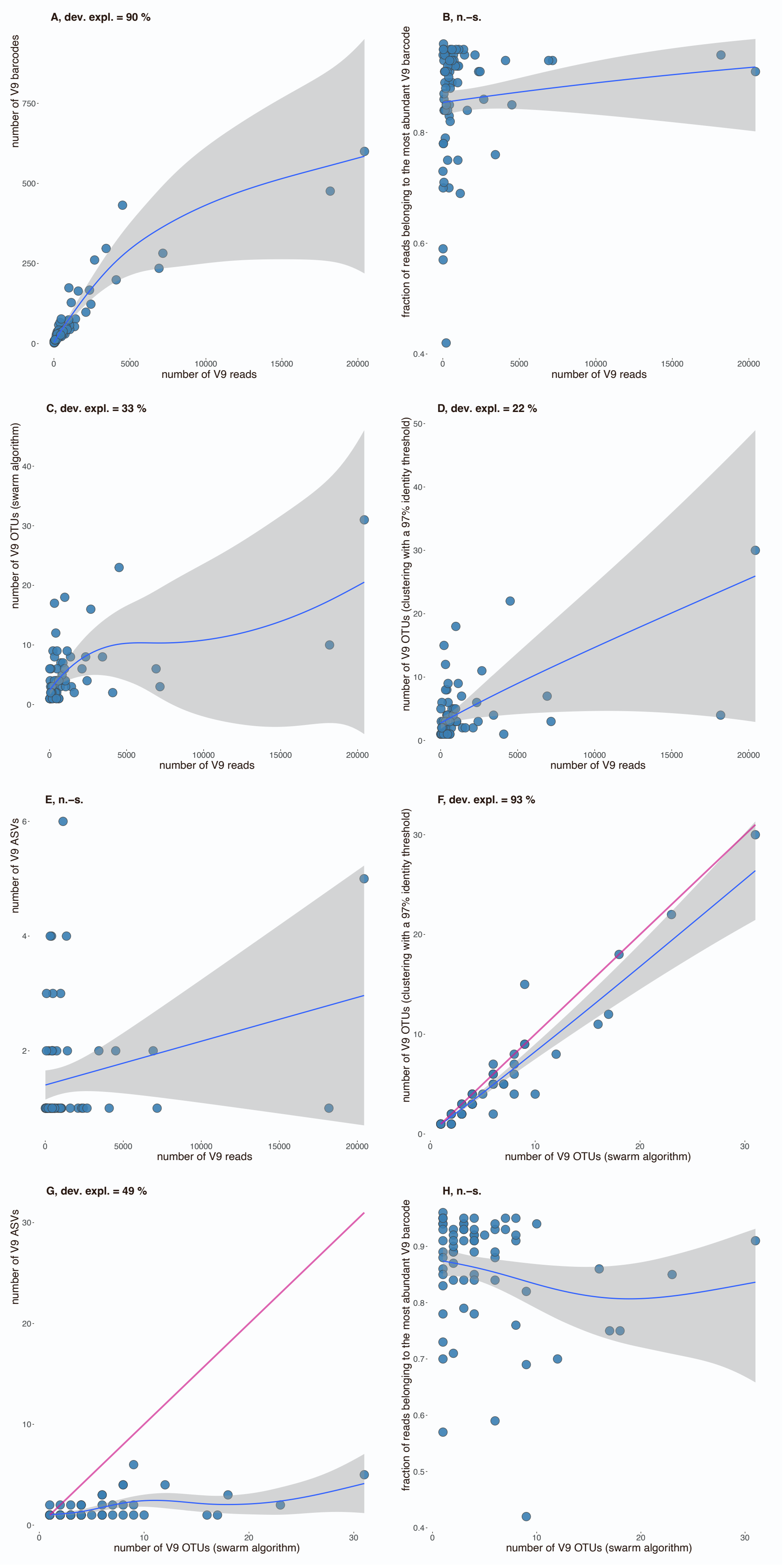

A, dev. expl. = 90 %
number of V9 barcodes
number of V9 reads
B, n.–s.
fraction of reads belonging to the most abundant V9 barcode
number of V9 reads
C, dev. expl. = 33 %
number of V9 OTUs (swarm algorithm)
number of V9 reads
D, dev. expl. = 22 %
number of V9 OTUs (clustering with a 97% identity threshold)
number of V9 reads
E, n.–s.
number of V9 ASVs
number of V9 reads
F, dev. expl. = 93 %
number of V9 OTUs (clustering with a 97% identity threshold)
number of V9 OTUs (swarm algorithm)
G, dev. expl. = 49 %
number of V9 ASVs
number of V9 OTUs (swarm algorithm)
H, n.–s.
fraction of reads belonging to the most abundant V9 barcode
number of V9 OTUs (swarm algorithm)

Figure S2. Dependence of the number of V9 barcodes (**A**), of the fraction of reads belonging to the most abundant V9 barcode (**B**), and of the number of V9 OTUs/ASVs defined using different methods (**C, D, E**) on the number of V9 reads extracted from genomes/transcriptomes. Scatterplots illustrating correlations between the number of V9 OTUs defined by the *SWARM* algorithm and the number of OTUs defined by centroid clustering (**F**) or ASVs generated by the *DADA2* algorithm (**G**) are also shown. Another scatterplot illustrates correlation between the number of V9 OTUs defined by the *SWARM* algorithm and the fraction of reads belonging to the most abundant V9 barcode (**H**). The trends were approximated with generalized additive models and shown as blue lines, with 95% confidence intervals shown in grey. The percent of variance explained by generalized additive models is shown above the plots. Identity lines $y = x$ in panels **F** and **G** are shown in magenta.

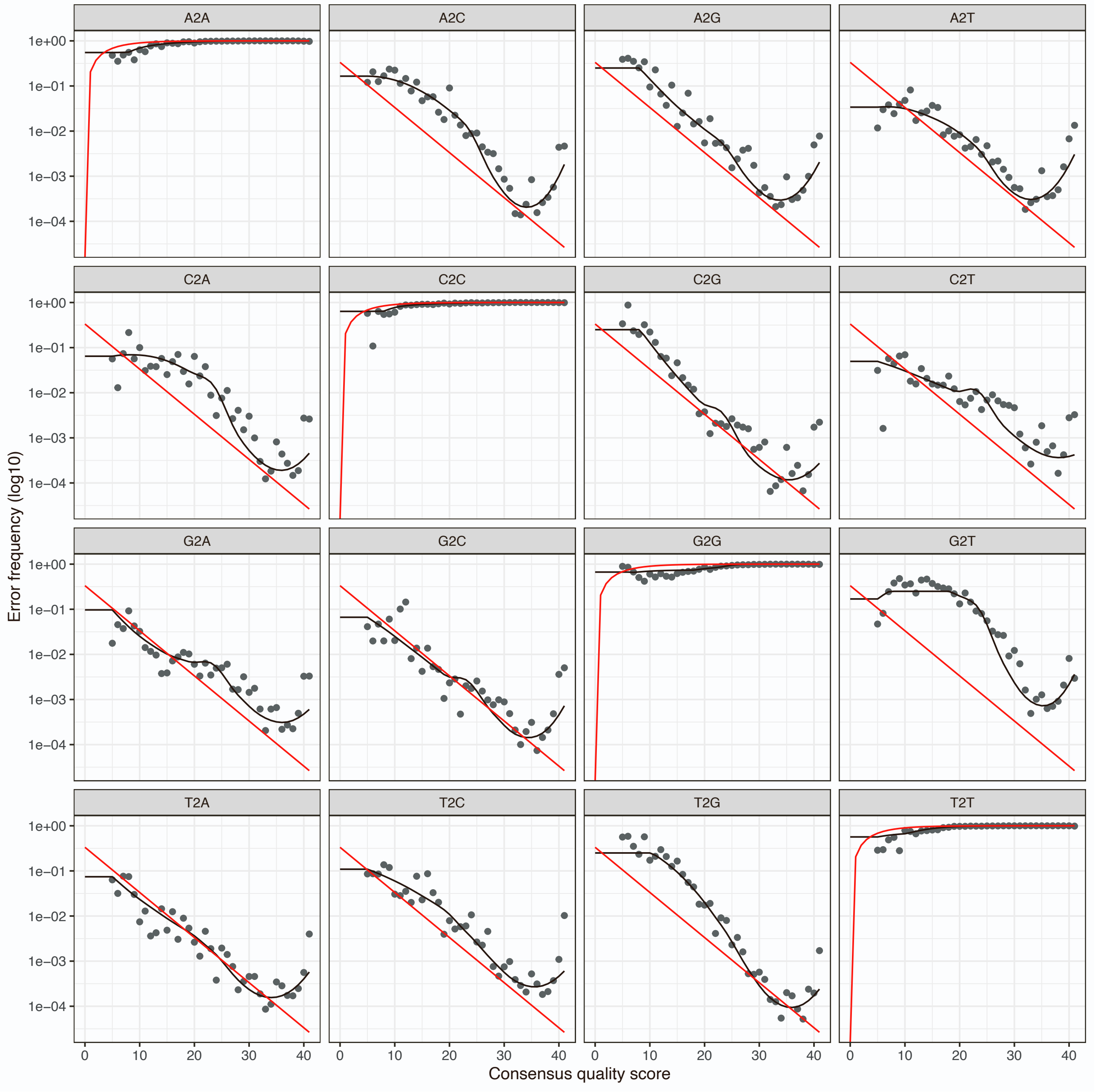

Figure S3. Error rates observed when applying the *DADA2* algorithm to forward reads from sample ERR562372 (the *Tara Oceans* V9 metabarcoding dataset). The observed error rates averaged across reads are shown on the y-axis, and Phred quality scores on the x-axis. Scatterplots for all possible mutation types (A→C, A→G, …) are shown. The black lines show the estimated error rates after convergence of the machine-learning algorithm. The red lines show the error rates expected under the nominal definition of the Phred quality score.

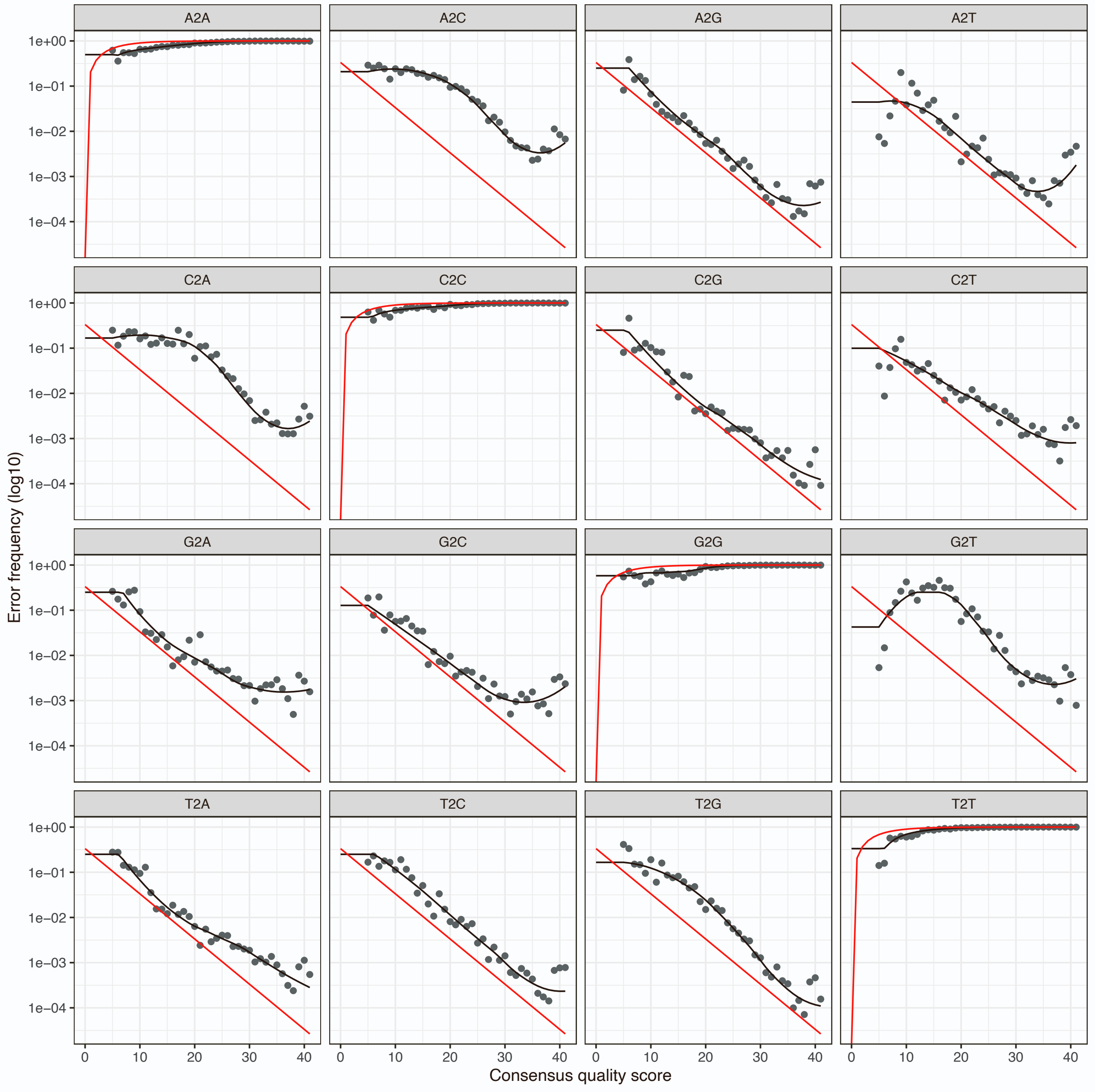

Figure S4. Error rates observed when applying the *DADA2* algorithm to reverse reads from sample ERR562372 (the *Tara Oceans* V9 metabarcoding dataset). The observed error rates averaged across reads are shown on the y-axis, and Phred quality scores on the x-axis. Scatterplots for all possible mutation types (A→C, A→G, …) are shown. The black lines show the estimated error rates after convergence of the machine-learning algorithm. The red lines show the error rates expected under the nominal definition of the Phred quality score.

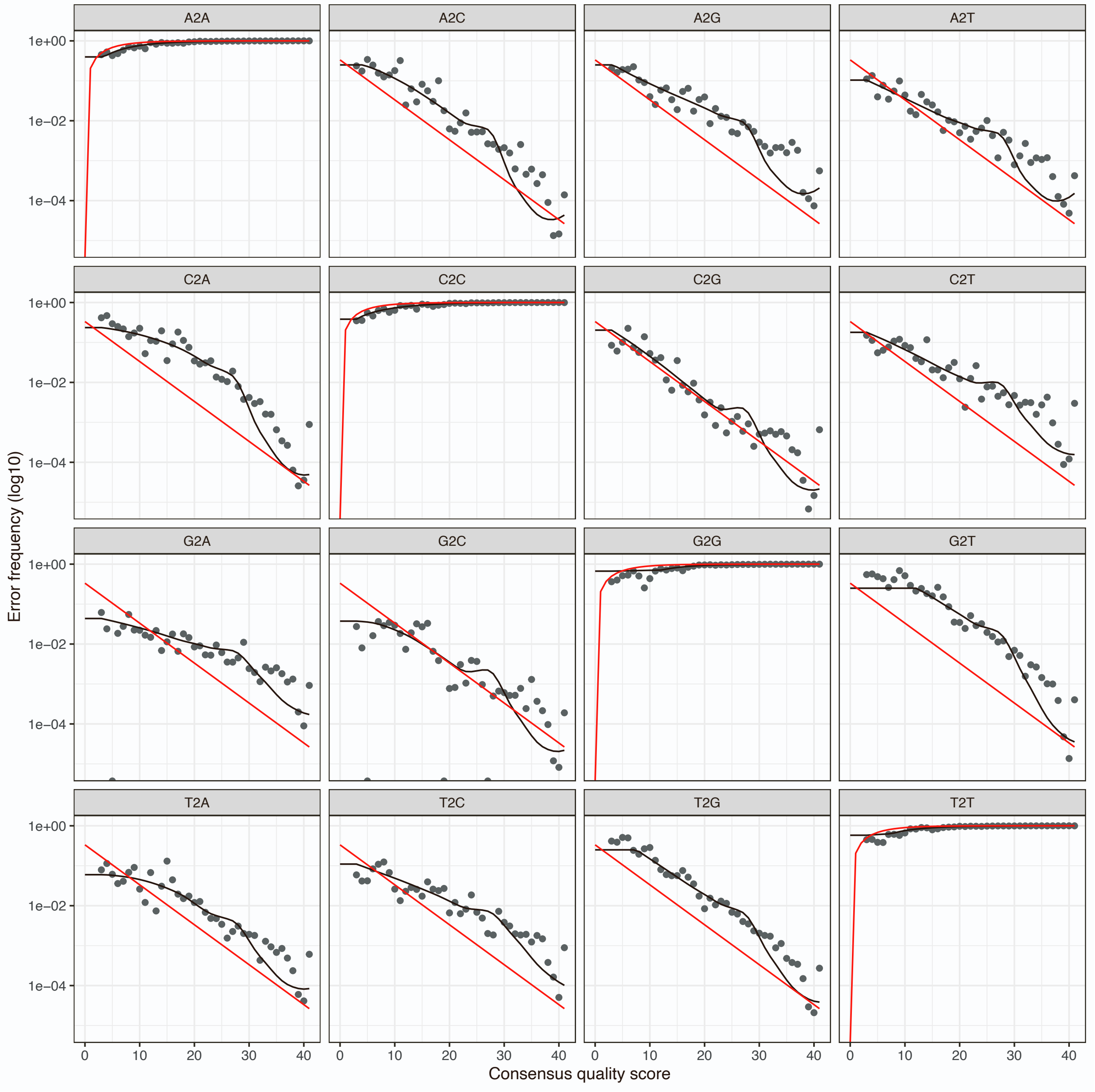

Figure S5. Error rates observed when applying the *DADA2* algorithm to merged reads from sample ERR562372 (the *Tara Oceans* V9 metabarcoding dataset). The observed error rates averaged across reads are shown on the y-axis, and Phred quality scores on the x-axis. Scatterplots for all possible mutation types (A→C, A→G, …) are shown. The black lines show the estimated error rates after convergence of the machine-learning algorithm. The red lines show the error rates expected under the nominal definition of the Phred quality score.

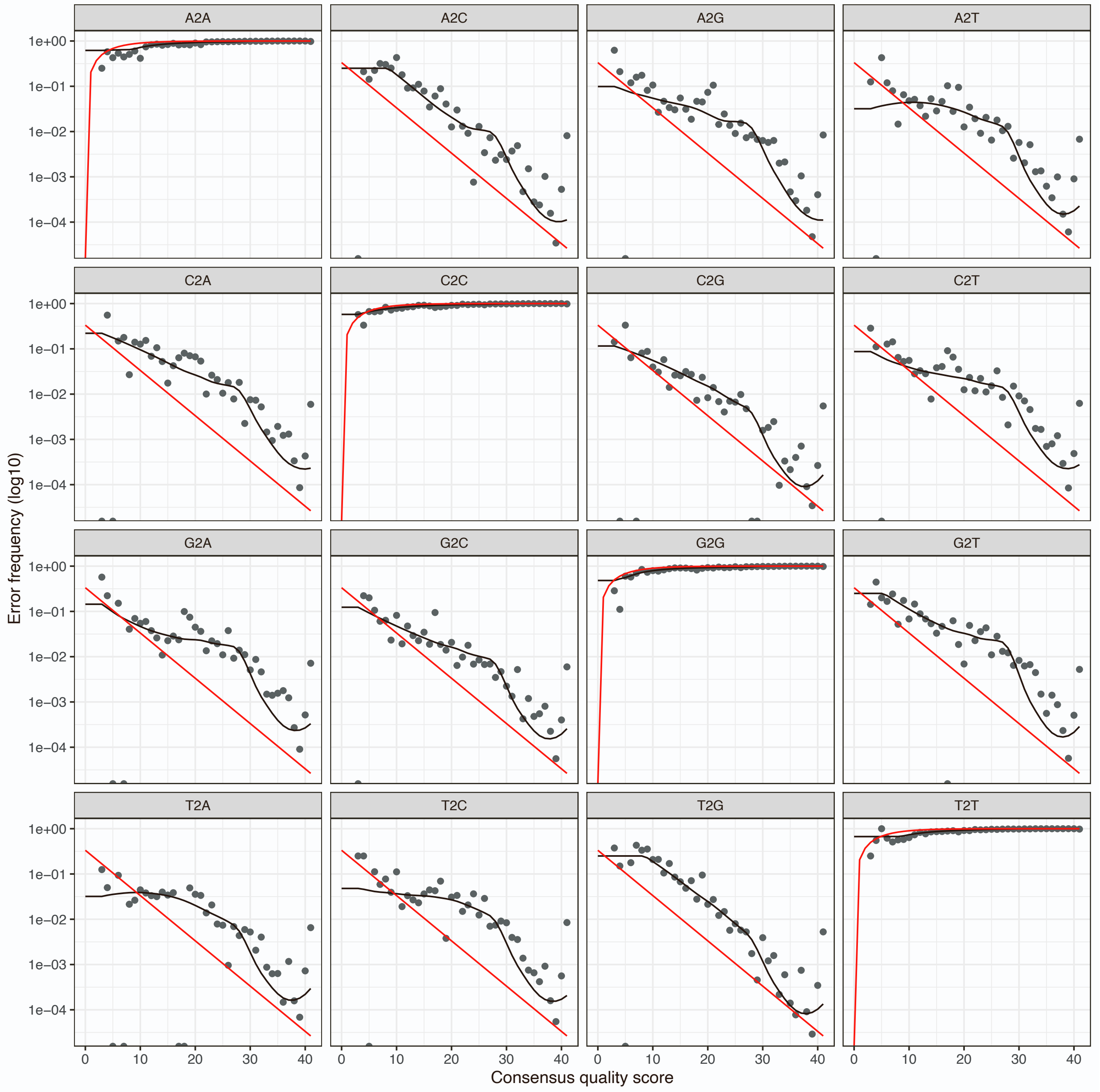

Figure S6. Error rates observed when applying the *DADA2* algorithm to pooled V9 regions extracted from the 68 genomes/transcriptomes. The observed error rates averaged across reads are shown on the y-axis, and Phred quality scores on the x-axis. Scatterplots for all possible mutation types (A→C, A→G, …) are shown. The black lines show the estimated error rates after convergence of the machine-learning algorithm. The red lines show the error rates expected under the nominal definition of the Phred quality score.

Supplemental table legends

Table S1. List of all V9 18S rRNA barcodes extracted from the 68 genomes/transcriptomes. The table contains the following information: genome/transcriptome ID (run ID), V9 barcode ID, sequence of the barcode, number of genomic/transcriptomic reads mapped to this barcode, number of reads mapped to a corresponding ASV (inferred by the *DADA2* algorithm), ID of a *SWARM* OTU the barcode belongs to, abundance (read number) of the *SWARM* OTU in the *Tara Oceans* metabarcoding data, percent similarity to a V9 barcode most abundant in the genome/transcriptome, percent similarity to a best-matching sequence in the reference database, taxonomic annotation of the best-matching reference sequence and the reference sequence itself.

Table S2. Data sources for the genomes/transcriptomes used in the study and statistics related to V9 reads and ASVs/OTUs. The results presented here were generated after contamination removal (see the main text and Methods).

Table S3. List of ASVs inferred in the 68 genomes/transcriptomes using the DADA2 denoising algorithm. Rows in the table are colored in the following way: ASVs predominant in two genome/transcriptome samples are highighted in blue-grey; ASVs predominant in one genome/transcriptome are highlighted in green; minor ASVs with a perfect match (100% similarity) to a V9 region sequence in the reference database are highlighted in pink (these ASV were interpreted as contamination and removed from further analyses); minor ASVs with <100% similarity to a best-matching sequence in the reference database are highlighted in yellow.

Table S4. Abundance of (supposedly) intragenomic ASVs and *SWARM* OTUs derived from them in the *Tara Oceans* metabarcoding data. Only ASVs passing the contamination removal step illustrated in Table S3 are shown here. Rows in the table are colored in the following way: ASVs predominant in two genome/transcriptome samples are highlighted in blue-grey; ASVs predominant in one genome/transcriptome are highlighted in green; minor ASVs with <100% similarity to a best-matching sequence in the reference database are highlighted in yellow.

Table S5. Diversity estimates for eukaryotic phyla in marine plankton based on the *Tara Oceans* V9 metabarcoding data and five ASV/OTU inference methods. ASVs and OTUs were inferred using five methods: *DADA2* on merged V9 amplicon reads (following the same protocol as applied to the genomic data); *DADA2* on non-merged V9 amplicon reads (following the protocol from [9, 10]); *SWARM* OTUs derived from V9 barcodes; ASVs generated using both *DADA2* protocols were optionally clustered with *SWARM*. Barcodes demonstrating low similarity (<80%) to any sequence in

the reference database were removed from the analysis. The taxa shown here were defined according to Adl. et al., 2019 and are non-overlapping. Ratios of selected ASV/OTU counts are shown in the five columns on the right.

Table S6. A list of SRA accession numbers for the *Tara Oceans* V9 metabarcoding dataset used in this study.
